# Controlled Electron-Beam Synthesis of Transparent Hydrogels for Drug Delivery Applications

**DOI:** 10.3390/polym11030501

**Published:** 2019-03-14

**Authors:** Sarah Glass, Mathias Kühnert, Bernd Abel, Agnes Schulze

**Affiliations:** Leibniz Institute of Surface Engineering (IOM), Permoserstraße 15, D-04318 Leipzig, Germany; sarah.glass@iom-leipzig.de (S.G.); mathias.kuehnert@iom-leipzig.de (M.K.); bernd.abel@iom-leipzig.de (B.A.)

**Keywords:** electron beam, polymerization, hydrogels, PEDGA, crosslinking degree, drug delivery

## Abstract

In this study, we highlight hydrogels prepared by electron-beam polymerization. In general, the electron-beam-polymerized hydrogels showed improved mechanical and optical transmittances compared to the conventional UV-cured hydrogels. They were more elastic and had a higher crosslinking density. Additionally, they were transparent over a broader wavelength range. The dependence of the mechanical and optical properties of the hydrogels on the number of single differential and total irradiation doses was analyzed in detail. The hydrogels were prepared for usage as a drug delivery material with methylene blue as a drug model. In the first set of experiments, methylene blue was loaded reversibly after the hydrogel synthesis. Electron-beam-polymerized hydrogels incorporated twice as much methylene blue compared to the UV-polymerized gels. Furthermore, the release of the model drug was found to depend on the crosslinking degree of the hydrogels. In addition, electron-beam polymerization enabled the irreversible binding of the drug molecules if they were mixed with monomers before polymerization.

## 1. Introduction

Hydrogels are polymeric networks synthesized from hydrophilic monomers [1,2]. In general, various monomers and polymerization methods can be applied for this purpose [3,4]. Besides thermal initiation, a common method to initiate radical polymerization is UV radiation exposure [3,5,6]. Herein, a photoinitiator is required in the formulation. The photoinitiator forms radicals when irradiated with UV light. Subsequently, the radicals induce polymerization [7]. UV curing is a widespread method, such as in 3-D printing [8], tissue engineering [9], and material coating [10].

Nevertheless, UV polymerization has some disadvantages. One is the potential cytotoxicity of several photoinitiators [11,12,13]. For example, 1-hydroxycyclohexyl phenyl ketone and 2,2-dimethoxy-2-phenylacetophenone were found to be cytotoxic for several cell lines, even at low concentrations [11]. Also, photoinitiators can transform into toxic products through irradiation.

Therefore, focus has shifted to photoinitiator-free polymerization for the synthesis of biomaterials [8]. One option is electron-beam curing. In this method, electrons are directly employed to initiate radical reactions. Electron energy (typically in keV or MeV) affects the penetration depth of the electrons in the sample. The electrons can induce polymerization reactions without additional chemicals, solvents, or initiators. Additionally, the material is simultaneously sterilized during the electron-beam treatment. Therefore, the method may be considered to be environmentally friendly and is suitable for the engineering of biomedical materials [3]. Consequently, the electron-beam technology has been used to synthesize several biomaterials [14,15,16,17]. For instance, Mennillo et al. demonstrated the electron-beam-induced preparation of a free-standing ion-gel. The ion-gel was synthesized without additional solvents, and it was mechanically stable. The introduced method is scalable and compatible with roll-to-roll processes, and it is therefore suitable for large-scale production [17].

Usually, material properties depend strongly on the applied monomer and the polymerization method [18]. Additionally, in electron-beam polymerization, the number of single differentials and total irradiation doses can be used to tailor the crosslinking degree. Thus, the resulting material properties can be adjusted with high precision [19,20].

In this study, electron beam technology was used for the synthesis of hydrogels. The crosslinking degree of the hydrogels was controlled by adjusting the number of single differentials and total irradiation doses. For comparison, a “standard” hydrogel prepared by UV polymerization of the same monomer was used [21]. Furthermore, the suitability of the hydrogel for medical application was characterized. For this purpose, hydrogels were loaded with the photoactive drug model methylene blue. Photosensitizers produce antimicrobial singlet oxygen when irradiated with light, and are applied in photodynamic therapy [22,23,24]. In former studies, a reversible loading of photosensitizers was investigated in UV-cured hydrogels [21,25]. Previously, irreversible immobilization of photoactive drug was not possible using UV polymerization [21]. Methylene blue inhibits most photoinitiators by absorbing light in the same spectral range. Electron-beam polymerization can overcome this issue. However, the use of electron-beam irradiation enables both reversible loading and irreversible immobilization within the hydrogel matrix. The irreversible binding of small molecules on polymeric surfaces was previously reported [26,27]. To our best knowledge, in this study methylene blue was irreversibly immobilized in a hydrogel using electron beam technology for the first time . Thus, the hydrogels can be synthesized for different applications, allowing or preventing the release of the photoactive drug. Both application routes can be followed by electron-beam technology for hydrogel preparation.

## 2. Materials and Methods

### 2.1. Materials

Poly(ethylene glycol) diacrylate (PEGDA) with an average molar mass of 700 g·mol^−1^, phosphate-buffered saline (PBS), and methylene blue were purchased from Sigma Aldrich (St. Louis, MI, USA). The photoinitiator 1-[4-(2-hydroxyethoxy)phenyl]-2-hydroxy-2-methyl-1-propan-1-one (*α*-HAP) was purchased from IGM Resins (Beijing, China). All chemicals were used without further purification. Water was purified using a Merck ultrapure water system (Burlington, VT, USA).

### 2.2. Preparation of the UV-Cured Hydrogels

The UV-cured hydrogels were synthesized as reported previously [21]. A formulation containing 30 wt % PEGDA, 0.1 wt % α-HAP, and PBS was prepared. One milliliter of the formulation was injected in a polystyrene mold of 35 mm in diameter. The formulation was polymerized using a medium-pressure mercury lamp. The dose was set to 2500 mJ·cm^−2^. The irradiation duration was 0.5 s. No significant temperature increase was detected during the irradiation. The resulting gels had a thickness of 1 mm and were washed twice for one hour in PBS (pH = 7.4). Afterward, the hydrogels were washed three times in milli-Q water and dried for 24 h at 40 °C. The mass of the resulting swollen hydrogels was 1 mg. The UV-cured samples were used as a reference and are abbreviated as “UV” henceforth.

### 2.3. Preparation of the Electron-Beam-Cured Hydrogels

For the preparation of the electron-beam-cured hydrogels, a formulation containing 30 wt % PEGDA in PBS was fabricated and injected in polystyrene molds. No photoinitiator was used. However, this time, the formulation was polymerized using a 10 MeV linear accelerator (MB10-30 MP, Mevex Corp, Stittville, ON, Canada). The total and differential doses were varied to investigate the influence of the crosslinking procedure on the resulting hydrogels. The washing and drying procedures were similar to those described in Section 2.2. The irradiation duration was 1.05 s for the samples with 3 kGy differential dose, and 2.1 s for the samples prepared with 6 and 30 kGy, respectively. No significant temperature increase was detected during the irradiation. The resulting gels had a thickness of 1 mm.

The hydrogels were labeled as “EB differential dose/total dose,” for example, “EB 3/30” was cured using a total dose of 30 kGy in 10 × 3 kGy steps. The following doses were used: 3/3, 6/6, 6/12, 6/18, 6/24, 3/30, 6/30, 30/30.

### 2.4. Methylene Blue Loading after Polymerization

After polymerization of the hydrogels, methylene blue loading was performed by immersing the hydrogels in a 1 × 10^−4^ M solution of methylene blue. The hydrogels were immersed for 48 h in 10 mL of methylene blue solution.

### 2.5. Methylene Blue Immobilization by Polymerization

A hydrogel formulation of 30 wt % PEGDA containing 1 × 10^−4^ M methylene blue was prepared. Methylene blue was added as a solid powder to the formulation. The formulation was injected into the polystyrene molds and polymerized using a 10-MeV linear accelerator. The polymerization parameters, washing, and drying procedures were similar to those described in Section 2.2.

The hydrogels were labeled as “MBEB differential dose/total dose.” For example, “MBEB 3/30” contained methylene blue and was cured using a total dose of 30 kGy in 10 × 3 kGy steps. The following doses were used: 3/3, 6/6, 6/12, 6/18, 6/24, 3/30, 6/30, 30/30.

### 2.6. Thermal Analysis

Thermal stability tests were performed using thermogravimetric analysis. A Pyris 1 TGA (Perkin Elmer, Waltham, MA, USA) was used. The temperature range was 20–800 °C with a heating rate of 10 °C∙min^−1^. Air was used as purge gas.

Furthermore, differential scanning calorimetry (DSC) was used to determine the glass transition temperature (T_g_) of the hydrogels. DSC was performed using a DSC 8500 (Perkin Elmer, Waltham, MA, USA). The temperature range was −100 to 50 °C. Helium was used as purge gas. The heating rate was 50 °C∙min^−1^.

### 2.7. Mechanical Analysis

The dynamic modulus, G*, and the loss factor, tan(δ), were determined using rheology. This was performed using an MCR300 rheometer (Anton Paar, Graz, Austria) at 25 °C. The rheometer was equipped with a 25-mm probe head. The probe head was pressed on the sample with 10 N. The hydrogels were investigated in the dry state and had a diameter of 23 mm. All values were recorded at 1 Hz.

### 2.8. UV/Vis Analysis

The transmittance of the dried hydrogels was investigated using a UV-2101PC UV–VIS spectrometer (Shimadzu, Kyoto, Japan). The studied range was set to 200–800 nm with a 0.5-nm step width. Samples had an average thickness of 1 mm.

### 2.9. Methylene Blue Loading

The dried hydrogels were immersed in 10 mL of a 1 × 10^−4^ M methylene blue solution per hydrogel for 48 h. The absorbance of the solution at 660 nm was measured before and after immersion using an infinite M200 reader from TECAN (Maennedorf, Switzerland). A 1 × 10^−4^ M methylene blue solution was used as reference.

The concentration was used to calculate the mass of methylene blue (m) according to the following relation:

(1) m=c∗V∗M,

where c is the determined concentration, V is the volume of the solution for immersion (10 mL), and M is the molar mass of methylene blue (319.9 g·mol^−1^). The mass of incorporated methylene blue was calculated using:

(2) m (incorporated)=m (before immersion)−m (after immersion).

### 2.10. Methylene Blue Release

The dried loaded hydrogels were immersed in 10 mL PBS (pH = 7.4) buffer. The buffer was changed after 1, 2, 3, 4, 5, 6, 8, 24, 48, and 72 h. The concentration of methylene blue in the PBS buffer was determined using an infinite M200 reader from TECAN. The mass was calculated using Equation (1).

### 2.11. Calculation of Crosslinking Density

The crosslinking density of the hydrogels was calculated using the following equation [28]:

(3) $$v_{c}=\;\frac{G^{*}}{\mathrm{RT}}\;,$$

where G* is the dynamic modulus determined by rheology, R is the universal gas constant (8.314 J∙mol^−1^·K^−1^), and T is the temperature during the measurement (298 K).

## 3. Results and Discussion

### 3.1. Optical Transmittance

Because the hydrogels prepared in this study are intended for use as carriers for photoactive substances, their optical transparency is essential. To ensure that the photoactive substance can be activated by illumination, the matrix has to be transparent in the visible range.

**Figure 1:**
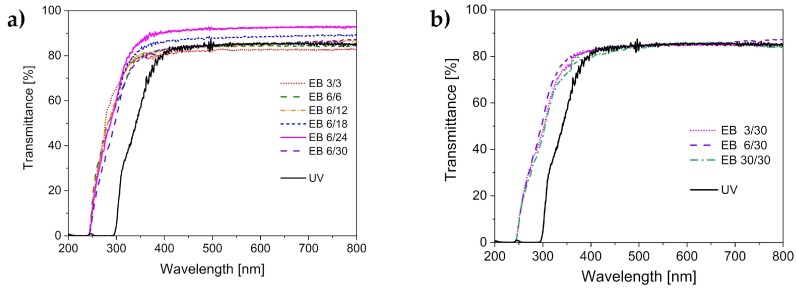
UV–Vis transmittance spectra of the UV-cured hydrogels compared to the hydrogels cured with electron beam (EB), with the variation of (**a**) the total doses and (**b**) the differential doses.

Figure 1 displays the transmittance of the hydrogels cured by UV and by electron-beam irradiations. The electron-beam-cured hydrogels possessed transparency within a broader wavelength range. Hydrogels cured with UV light were transparent at wavelengths greater than 300 nm. In comparison, electron-beam-cured hydrogels were already transparent with wavelengths above 250 nm. We assume that the presence of the photoinitiator caused the higher cut-off wavelength for the UV-polymerized hydrogels. α-HAP has an absorption maximum at 280 nm. Thus, its absence enables the application of the electron-beam-polymerized hydrogels at smaller wavelengths.

The value of the transmittance was comparable for both types of hydrogels and did not seem to depend on the irradiation technique. However, minor variations were observed, related to the irradiation dose applied to the hydrogel formulation. In the electron-beam treatment, the application of a total dose of 18 or 24 kGy led to hydrogels with 10% higher transparency compared to the application of smaller doses or UV irradiation. This effect may be explained by the lower defect density (e.g., air inclusions, microcracks) in hydrogels cured with higher doses of the electron beam. Presumably, a higher crosslinking degree (see also Section 3.3) was associated with fewer defects. However, there was an optimal dose, since the application of a larger total irradiation dose of 30 kGy resulted in a further reduction of transparency. This can be explained by the high concentration of radical species formed by the high irradiation dose. With the increase in the concentration of radicals, the chance of recombination reactions increases. Consequently, the crosslinking density decreases when a specific total dose is exceeded, and this finally affects the transparency of the hydrogel.

### 3.2. Thermal Stability

**Figure 2:**
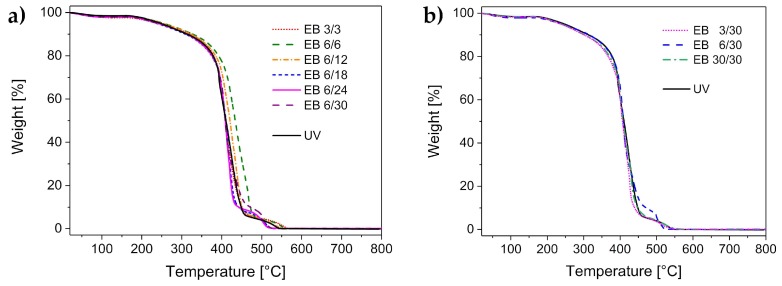
Thermal stability of the UV-cured hydrogels compared to the hydrogels cured with electron beam, with the variation of (**a**) the total doses and (**b**) the differential doses.

Appropriate thermal stability of the hydrogels enables sterilization via autoclaving, which is a widely used method to sterilize medical products. It is usually performed within the temperature interval of 120–134 °C. The results of the thermal stability analysis are displayed in Figure 2. The thermal stability was found to be comparable for all investigated hydrogels. All samples had a small weight loss at 100 °C, which was related to the loss of water. The hydrogels were dried and stored in air before performing the experiments. Thus, a slight amount of water was left in the samples because of the ambient humidity. The water loss amounted to 2%–4%.

The hydrogels were stable up to 170 °C. Since autoclaving is performed at lower temperatures, the hydrogel sterilization is ensured.

The hydrogels started to degrade at temperatures higher than 170 °C. The degradation took place in several steps, and was completed at 530 °C. The degradation profile was similar for both the UV-cured and the electron-beam-cured hydrogels.

### 3.3. Mechanical Properties

The crosslinking degree of a polymer significantly impacts its mechanical properties. The glass transition temperature, the dynamic modulus G*, and the loss factor tan(δ) were determined in order to characterize the crosslinking. Usually, the glass transition temperature is higher with increasing polymer chain length [29]. Thus, the glass transition temperature is higher in the case of higher crosslinking densities. The crosslinking degree also impacts the dynamic modulus (G*). The dynamic modulus is composed of the storage modulus and the loss modulus. A higher G* value is correlated with a higher crosslinking density [30]. Finally, the viscoelastic properties of a material can be described by the loss factor, tan(δ). The lower the value of tan(δ), the more prominent are the elastic properties.

**Figure 3:**
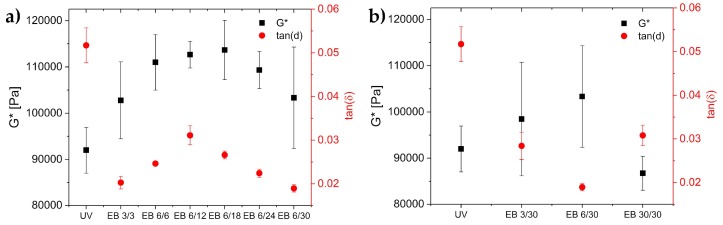
Dynamic modulus (G*) and loss factor (tan(δ)) of the UV-cured hydrogels compared to the hydrogels cured with electron beam, with the variation of (**a**) the total doses and (**b**) the differential doses. All data were recorded at 1 Hz.

G* and tan(δ) values of the hydrogels are displayed in Figure 3. G* was higher for the hydrogels cured with electron beam (100–120 kPa) compared to UV-cured hydrogels (90 kPa). Hence, the crosslinking density was higher in the hydrogels cured by electron beam compared to those cured by UV (Figure 3a). However, exposing the hydrogels to 30 kGy in a single irradiation step resulted in a lower G* (85 kDa), as shown in Figure 3b. This indicated a lower crosslinking density as compared to the other irradiation experiments.

The crosslinking densities of all hydrogels were calculated using Equation (3). The values are displayed in Table 1. The crosslinking density of the UV-polymerized hydrogels was 37.1 mmol·L^−1^, while the electron-beam-cured hydrogels had crosslinking densities of 35.0–45.9 mmol·L^−1^. Obviously, the crosslinking density reached the highest value for hydrogels prepared with 6 kGy differential doses and total doses of 6 to 24 kGy. Except for the hydrogels prepared with 30 kGy in a single irradiation step, all electron-beam-polymerized hydrogels had crosslinking densities higher than those of the UV-polymerized hydrogels. 

Compared to the UV-cured hydrogel, the loss factor of the electron-beam-cured hydrogels was found to be divided by two (UV:tan(δ) = 0.055; electron beam:tan(δ) = 0.02–0.03). Accordingly, the elastic properties of the electron-beam-derived hydrogels were increased compared to the UV-cured gels. We assumed that the elastic properties increased with increasing crosslinking degree, as formerly described in Section 3.1.

**Figure 4:**
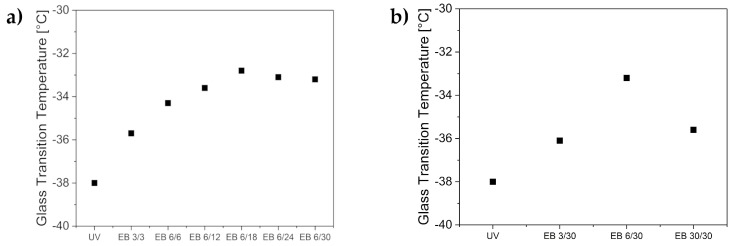
Glass transition temperatures of the UV-cured hydrogels compared to the hydrogels cured with electron beam, with the variation of (**a**) the total doses and (**b**) the differential doses.

Additionally, the glass transition temperature was investigated. The results are displayed in Figure 4. The glass transition temperature of the UV hydrogel was determined to be −38 °C. The electron-beam-cured hydrogels had a higher glass transition temperature of −36 to −33 °C. These results again indicate a higher degree of crosslinking for the electron-beam-cured hydrogels. Additionally, the glass transition temperature was observed to reach a plateau at −33 °C when the hydrogels were prepared with a total irradiation dose of more than 18 kGy. Thus, the crosslinking density increased with increasing total irradiation dose. Again, the single irradiation impact was investigated, and the EB 30/30 hydrogel resulted in lower crosslinking density. This result supports previous assumptions. Consequently, electron-beam irradiation with the total irradiation doses of 12 to 18 kGy using 6 kGy differential doses led to the hydrogels with the highest crosslinking density and highest mechanical stability.

Additionally, IR spectra were recorded (Electronic Supplementary Information, Figure S1). All hydrogels showed near-complete double-bond conversion. The IR signal at 1639 cm^−1^ indicating the acrylic double bond was significantly reduced.

### 3.4. Loading and Immobilization of Methylene Blue in Hydrogels

**Figure 5:**
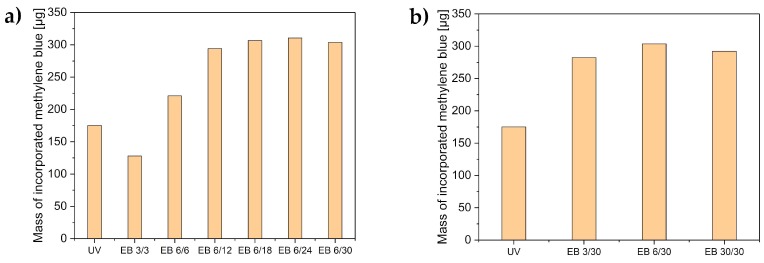
Methylene blue uptake of the UV-cured hydrogels compared to the hydrogels cured with electron beam, with the variation of (**a**) the total doses and (**b**) the differential doses.

The hydrogels were loaded with methylene blue either before or after polymerization. The mass of methylene blue in the hydrogels was determined using Equations (1) and (2). The results are displayed in Figure 5.

All electron-beam-polymerized hydrogels (except EB 3/3) incorporated more methylene blue than the UV-polymerized hydrogels. Hydrogels prepared at higher total doses contained the most methylene blue. All hydrogels with a total dose exceeding 18 kGy took up more than 300 µg of methylene blue. This corresponded to a complete uptake (98%) of methylene blue from the immersion solution (316 µg). Higher crosslinking led to a higher methylene blue uptake. Effective uptake of the model drug will enable a higher loading and therefore, higher release quantities.

Additionally, methylene blue was irreversibly immobilized with electron-beam polymerization by adding 1 × 10^−4^ M methylene blue to the monomer formulation. Thus, methylene blue was irreversibly encapsulated during the polymerization. The thus-prepared hydrogels incorporated a quantity of 32 µg (1 × 10^−4^ M) methylene blue.

### 3.5. The Release of Methylene Blue

**Figure 6:**
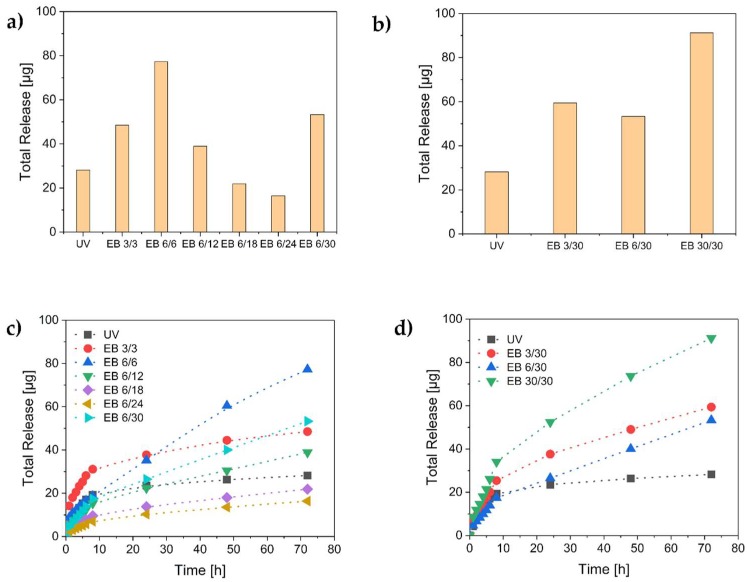
The release of methylene blue from the hydrogels. Total release of the hydrogels with the variation of (**a**) the total doses and (**b**) the differential doses. Time-dependent release behavior of (**c**) the total doses and (**d**) the differential doses.

The release of methylene blue from the hydrogels was monitored for 72 h. First, the results of the methylene blue release after the reversible hydrogel loading were observed (Figure 6).

The total release of methylene blue from the electron-beam-polymerized hydrogels was equal to or higher than the release from the UV-polymerized ones. Obviously, the degree of crosslinking influenced the release behavior of the hydrogels. The electron-beam-polymerized hydrogels with the highest crosslinking density showed the lowest release of methylene blue (Figure 6a). Additionally, the methylene blue release of the UV-crosslinked hydrogels was completed within 24 h. However, a longer release period was observed in the case of the electron-beam hydrogels (Figure 6c,d). The releases of the hydrogels EB 6/30, EB 30/30, and EB 6/6 were not completed after 72 h. Accordingly, even longer release periods would be possible. Therefore, the electron-beam-polymerized hydrogels enable long-term applications. These findings indicate that the release time depends on the crosslinking density of the hydrogel. A low crosslinking density was correlated with a high long-term release. In contrast, a high crosslinking density caused a slow release of small amounts of methylene blue, as in the case of EB 6/18 and EB 6/24. Lower release was correlated with higher uptake (see Section 3.4). Presumably, a higher degree of crosslinking generates a larger inner surface. Therefore, more methylene blue was adsorbed onto the surface and was not released again.

**Figure 7:**
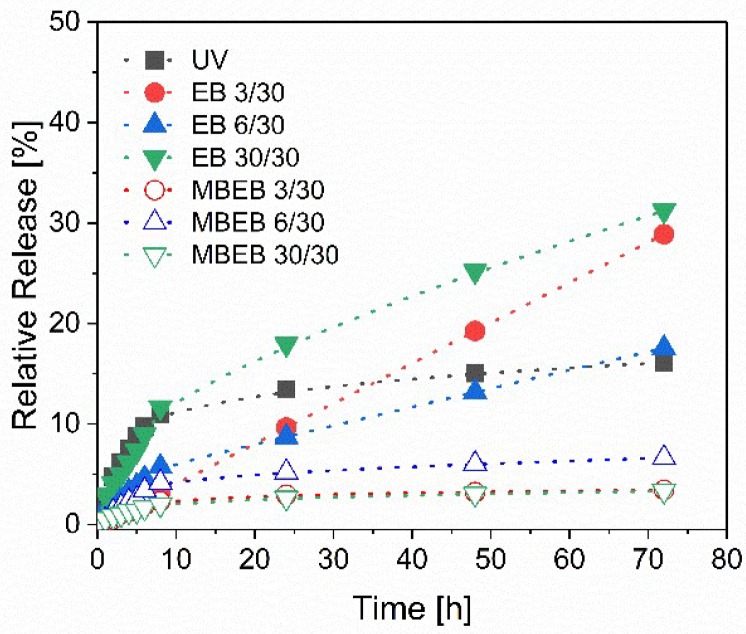
The methylene blue release of the hydrogels loaded reversibly (filled symbols) and irreversibly (empty symbols). MBEB: hydrogels with methylene blue incorporated before polymerization.

Furthermore, hydrogels loaded with methylene blue in the polymerization process and partially covalently linked to the polymer network were investigated. When the hydrogels were prepared for reversible methylene blue loading, the highest release was found after the synthesis with a total dose of 30 kGy. Therefore, we used the same dose for the hydrogel synthesis and irreversible methylene blue loading. The results are displayed in Figure 7.

The hydrogels with methylene blue incorporated before polymerization did not release comparable amounts of methylene blue (label MBEB) to the previously described hydrogels. The hydrogel MBEB 30/30 released only 1 µg methylene blue after 72 h (=3% of the incorporated methylene blue). In contrast, the hydrogel EB 30/30 released 91 µg (31%). Therefore, an irreversible immobilization of the model drug methylene blue was possible with electron-beam polymerization.

In conclusion, tailored variations of the electron-beam treatment enable the design of tailor-made hydrogels for specific or individual applications.

## 4. Conclusions

This study demonstrates clear advantages of electron-beam compared to UV polymerization. The hydrogels prepared by electron-beam polymerization had a higher crosslinking density and were more stable than the UV-polymerized ones. Additionally, they were transparent in a broader wavelength range due to the absence of a photoinitiator. In general, electron-beam doses of 12–18 kGy provided the highest crosslinking density. Furthermore, applying several single differential doses led to higher crosslinking densities as compared to applying the total dose at once.

The electron-beam-polymerized hydrogels incorporated more model drug than the UV-cured hydrogels. Therefore, the hydrogels can be used not only as a carrier system but also as an absorber material. Electron-beam polymerization allows the preparation of tailor-made hydrogels for individual applications. Depending on the irradiation parameters, the loading capacity and release time can be adjusted. The underlying cause for this phenomenon is probably an increased inner surface. This assumption has to be examined in further studies. Furthermore, the electron-beam method enables the choice between reversible or irreversible loading of drugs.

Hydrogels with an irreversibly immobilized photosensitizer enable new applications, such as antimicrobial or antifouling surfaces. In conclusion, electron-beam irradiation was demonstrated to be a powerful tool to control biomaterials such as hydrogels.

## Figures and Tables

**Table 1:** Crosslinking density values calculated from the dynamic moduli.

	UV	EB 3/3	EB 6/6	EB 6/12	EB 6/18	EB 6/24	EB 6/30	EB 3/30	EB 30/30
G* (kPa)	92	103	111	113	114	109	103	98	87,000
ν (mmol∙L^−1^)	37.13	41.48	44.80	45.47	45.88	44.13	41.71	39.74	35.01

## Supplementary Materials

The following are available online at https://www.mdpi.com/2073-4360/11/3/501/s1. Figure S1: FT-IR spectra of the hydrogels and of the formulation. Spectra were recorded measuring ATR-IR using a Vector II Fourier-transform infrared (FTIR) spectrometer fromBillerica, MA, USA). Spectra were normalizied to the IR band at 1705 cm^−1^.
