# Supplementary material for: Controlled Electron-Beam Synthesis of Transparent Hydrogels for Drug Delivery Applications
Source: Polymers (Basel). 2019 Mar 14;11(3):501. doi: 10.3390/polym11030501 (PMC6473313; doi:10.3390/polym11030501)
Supplement: Supplementary file 1 [file polymers-11-00501-s001.pdf]

polymers

Article

Controlled electron beam synthesis of transparent hydrogels for drug delivery applications

Electronic Supplementary Information

Sarah Glass [1], Mathias Kühnert[1], Bernd Abel[1] and Agnes Schulze [1,*]

[1] Leibniz Institute of Surface Engineering (IOM), Permoserstraße 15, D-04318 Leipzig; sarah.glass@iom-leipzig.de (S.G.); mathias.kuehnert@iom-leipzig.de (M.K.); bernd.abel@iom-leipzig.de (B.A.); agnes.schulze@iom-leipzig.de (A.S.)

* Correspondence: Agnes.Schulze@iom-leipzig.de; Tel.: +49-(0)341-235-2400

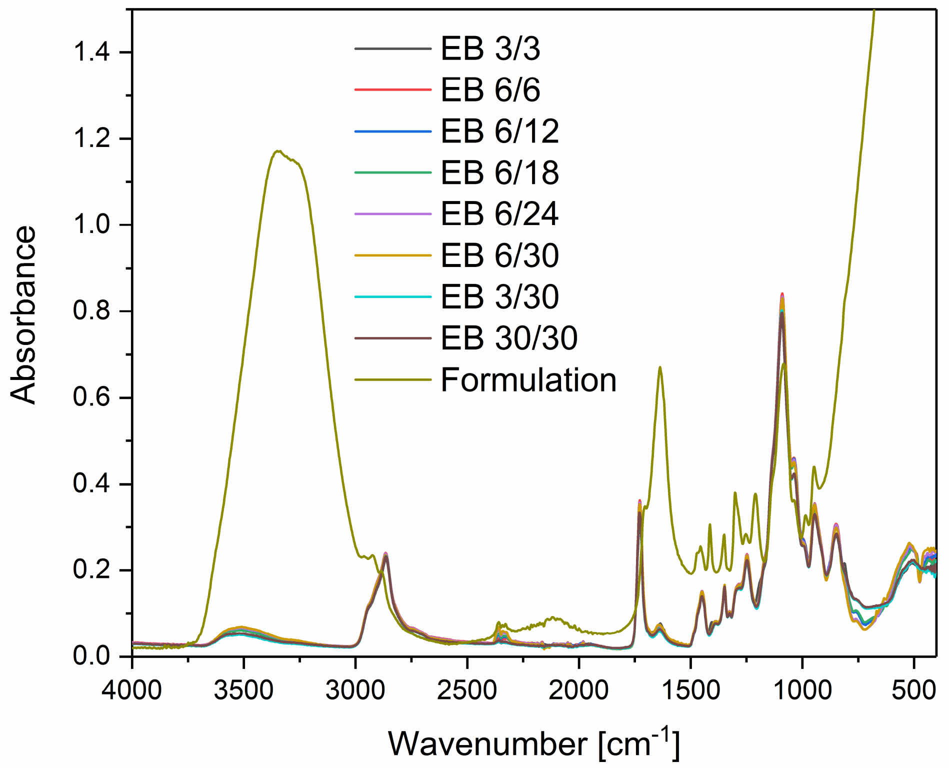

Figure S1: FT-IR spectra of the hydrogels and of the monomer formulation. Spectra were recorded measuring ATR-IR using a Vector II Fourier-transform infrared (FTIR) spectrometer fromBillerica, MA,USA). Spectra were normalizied to the IR band at 1705 cm^{-1}.
